# Atypical presentation of Wilms’ tumor in an adult: a case report and diagnostic considerations

**DOI:** 10.1093/jscr/rjae681

**Published:** 2024-11-06

**Authors:** Taha Yassine Aaboudech, Sabrine Derqaoui, Kaoutar Znati, Fouad Zouaidia, Ahmed Ibrahimi, Khalid Mzouri, Yassine Nouini, Zakia Bernoussi, Ahmed Jahid

**Affiliations:** Pathology Department, Ibn Sina Hospital, Rabat 10100, Morocco; Mohammed V University in Rabat, Rabat 10000, Morocco; Pathology Department, Ibn Sina Hospital, Rabat 10100, Morocco; Mohammed V University in Rabat, Rabat 10000, Morocco; Pathology Department, Ibn Sina Hospital, Rabat 10100, Morocco; Mohammed V University in Rabat, Rabat 10000, Morocco; Pathology Department, Ibn Sina Hospital, Rabat 10100, Morocco; Mohammed V University in Rabat, Rabat 10000, Morocco; Mohammed V University in Rabat, Rabat 10000, Morocco; Department of Urology A, Ibn Sina Hospital, Rabat 10100, Morocco; Mohammed V University in Rabat, Rabat 10000, Morocco; Department of Urology A, Ibn Sina Hospital, Rabat 10100, Morocco; Mohammed V University in Rabat, Rabat 10000, Morocco; Department of Urology A, Ibn Sina Hospital, Rabat 10100, Morocco; Pathology Department, Ibn Sina Hospital, Rabat 10100, Morocco; Mohammed V University in Rabat, Rabat 10000, Morocco; Pathology Department, Ibn Sina Hospital, Rabat 10100, Morocco; Mohammed V University in Rabat, Rabat 10000, Morocco

**Keywords:** adult, case report, histopathology, nephroblastoma, Wilms tumor

## Abstract

Wilms’ tumor (WT), while common in pediatric populations, is exceedingly rare in adults, accounting for only 0.5% of renal neoplasms. This report describes a 44-year-old patient diagnosed with stage II WT in the left kidney who underwent a radical nephrectomy, illustrating the diagnostic challenges encountered in adult cases. The patient’s symptoms and radiological findings were non-specific, complicating early detection. Histopathological analysis revealed a mixed-type tumor with a 60% blastemal component. Accurate histopathological evaluation is crucial for determining prognosis and guiding treatment. This case is rare due to its early-stage (stage II) diagnosis in an adult, which is almost unheard of in the literature, where most adult WT are advanced. This highlights the critical need for heightened clinical suspicion, early diagnostic efforts, and pivotal surgery-based treatment to improve outcomes in such rare cases.

## Introduction

Wilms’ tumor (WT), also referred to as nephroblastoma is the most common primary malignant renal tumor in children, accounting for ~5%–6% of childhood neoplasms. However, it is extremely rare in adults, making up only 0.5% of all renal neoplasms [1]. The incidence in adults is exceptionally low, with fewer than 0.2 cases per million people annually. Because of its rarity, WT is rarely considered in older patients, often leading to delayed diagnosis and management, and it typically presents at a more advanced stage [2]. Clinically and radiologically, it is indistinguishable from renal cell carcinoma (RCC), making its diagnosis unexpected and often overdue. Additionally, the rarity of adult WT results in delayed application of appropriate and risk-adapted treatments based on international pediatric oncology protocols. This delay contributes to historically poorer survival outcomes in adults compared to children with Wilms’ tumor [3].

## Case report

**Figure 1:**
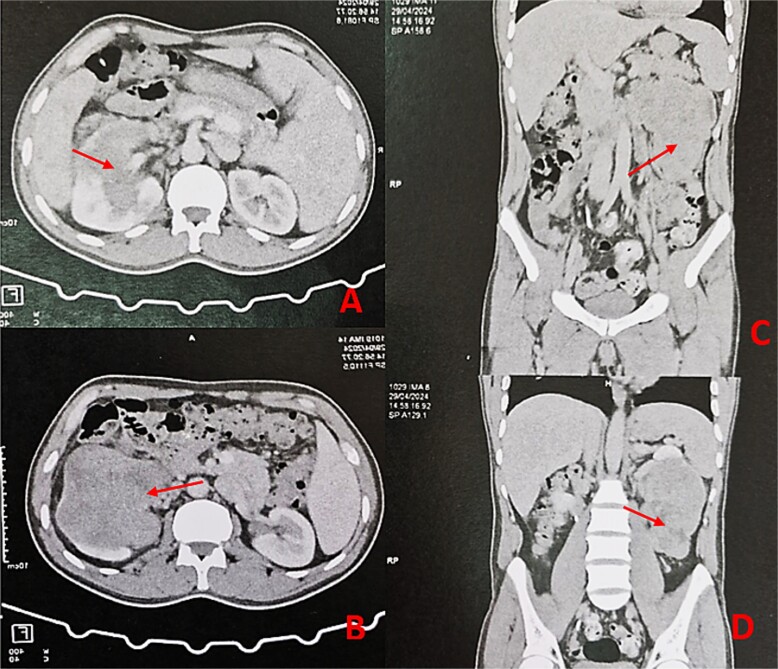
Thoraco-abdominopelvic CT scan with axial (A, B) and sagittal (C, D) images demonstrating a large heterogeneous mass (arrows) centered on the middle and lower thirds of the left kidney.

**Figure 2:**
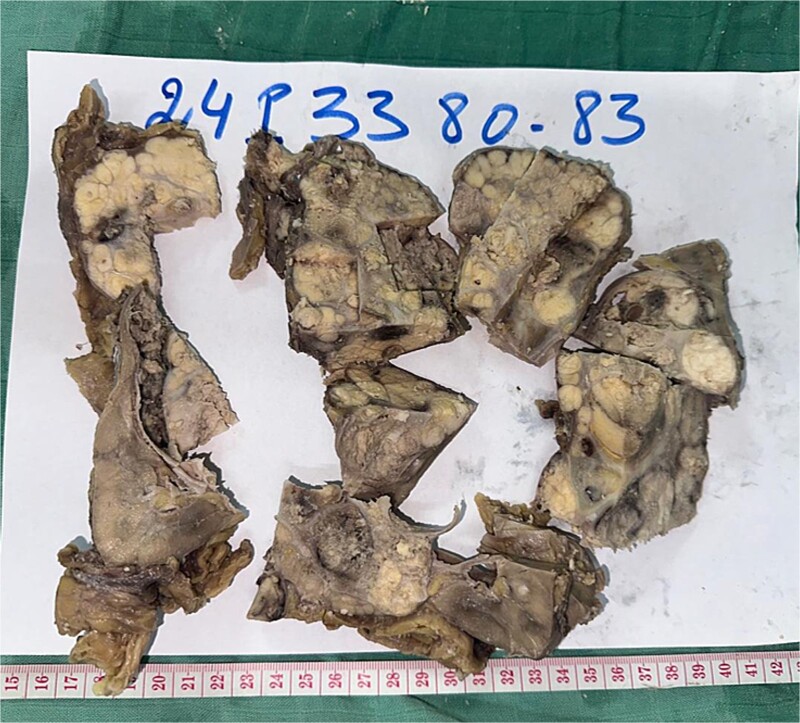
Radical nephrectomy reveals polylobulated white-yellow infiltrating tumor on gross examination.

A 44-year-old patient presents to a urologist with gross hematuria and left flank pain that has been ongoing for the last 4 months. An ultrasound was followed by a thoraco-abdominopelvic computed tomography (CT) scan, which revealed a large renal mass centered on the middle and lower third of the left kidney, infiltrating the entire calyceal system up to the left ureter. Additionally, there is perirenal fat infiltration (Fig. 1). The patient underwent a radical nephrectomy, and the specimen was sent to the pathology laboratory for histopathological examination. Gross examination of the radical nephrectomy specimen revealed a polylobulated, firm, white yellow tumor appearing to infiltrate the renal sinus and hilum, reaching the renal capsule without perirenal fat infiltration (Fig. 2). No macroscopic tumor embolus was observed in the renal vein. Lymphadenectomy of the perirenal fat revealed a lymph node measuring 1.1 × 0.8 × 0.5 cm.

**Figure 3:**
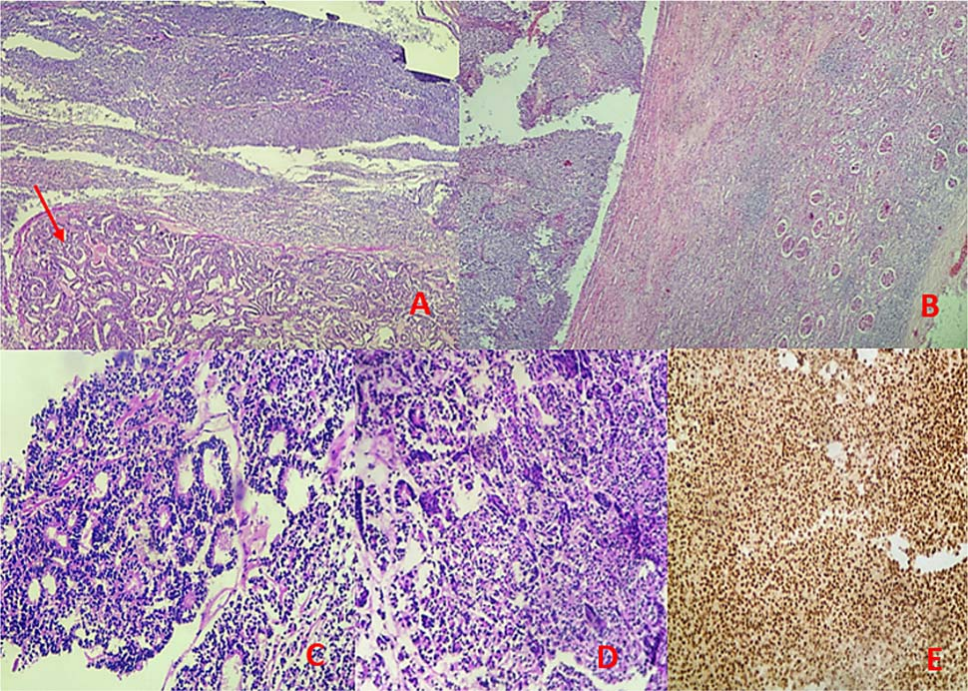
Microscopic findings: (A, B) hematoxylin–eosin (H&E) stain at low magnification reveals a mixed tumor with blastemal and epithelial components (arrow), showing well-defined borders pushing renal parenchyma. (C, D) Hematoxylin–eosin (H&E) stain at 40× magnification displays blastemal cells with scant cytoplasm, small size, mitotic activity, rounded overlapping nuclei, and primitive tubular structures. (E) Immunohistochemical analysis demonstrates diffuse nuclear staining for WT1.

Microscopic examination identified a triphasic WT with mixed components: 60% blastemal, 35% epithelial, and a very focal mesenchymal component. The tumor was classified as stage II according to the National Wilms’ Tumor Study (NWTS) Group staging system. There was no evidence of anaplasia or nephrogenic rests. The tumor infiltrated the renal sinus without lymph node metastasis. Immunohistochemical staining revealed positive expression for cytokeratin, WT1, and cyclin D1, while CD10, CK7, and AMARC were negative (Fig. 3).

## Discussion

WT is an embryonal neoplasm that originates from nephrogenic blastemal cells and mimics the histology of the kidneys, often showing various patterns of differentiation. This disease affects ~1 in every 7000 children. It occurs equally in both genders and in both kidneys, with the average age of onset being 35 months for males and 45 months for females. Notably, 98% of cases are diagnosed in children under the age of 10. While WT has been reported in adults, it remains extremely rare [4]. The clinical presentation of WT in adults differs from that in children. In adults, the main symptom is often flank pain, accompanied by a history of weight loss and a sudden decline in performance status. Occasionally, the tumor may be asymptomatic and detected incidentally through CT scans and ultrasound. In contrast, children typically present with a painless, palpable abdominal mass. Metastatic disease at diagnosis is more common in adults, occurring in 30% of cases compared with 10% in the pediatric population. Staging criteria of WT are based on the anatomic extent of the tumor at nephrectomy piece. There are two main classifications: NWTS, which proposes a pre-chemotherapy nephrectomy; and the International Society of Pediatric Oncology (SIOP) classification which advises four to six cycles of chemotherapy previous to nephrectomy. Up to 50% of adult cases are diagnosed at an advanced stage (III–V) [5]. Whereas our current case is classified as stage II with no lymph node or distant metastasis.

Kilton *et al.* [6] have established six strict diagnostic criteria for adult WT: it must be a primary renal tumor; it must have a primitive blastemal spindle or round cell component; it should form abortive or embryonal tubules or glomerular structures; the tumor must not show any areas diagnostic of RCC; there must be pictorial confirmation of histology; and the patient must be over 15 years old. Our case met all these criteria. Although rare, WT and RCC can occasionally be found simultaneously in the same kidney [1].

There is no histopathologic difference between adult and child WT. WT consists of blastemal, stromal, and epithelial components, present in varying proportions that influence prognosis. A predominance of blastemal components is associated with poor outcomes despite therapy, while epithelial and stromal components represent intermediate risk tumors [7].

Anaplasia, characterized by multipolar mitotic figures, enlarged nuclei at least three times the normal size, and hyperchromatic nuclei, is seen in ~10% of WT cases. Identifying anaplasia and describing its focal or diffuse distribution is crucial, as diffuse anaplasia is linked to higher relapse rates and worse outcomes [7].

Immunohistochemistry analysis is typically not required, but markers such as cytokeratin, vimentin, desmin, actin, and WT1 can help distinguish predominant blastemal tumors from rare ones. WT1 is expressed in some blastemal and epithelial elements but not in the stromal components [8].

Nephroblastoma is categorized into one of three risk groups based on histopathologic features, which is essential for determining appropriate therapeutic approaches. Blastemal-predominant WT are more aggressive and have a poorer prognosis, placing them in the high-risk group. Tumors with predominantly epithelial and stromal features are classified as intermediate risk. Tumors with a predominance of mesoblastic features fall into the low-risk group [7].

The staging criteria for WT are determined by the anatomical extent of the tumor. There are two main staging systems: the prechemotherapy, surgery-based system developed by NWTSG and the postchemotherapy-based system developed by SIOP. The NWTSG and the Children’s Oncology Group recommend resecting the primary tumor before administering chemotherapy. In contrast, SIOP advocates for administering chemotherapy for four weeks prior to surgery [3].

Although there are no established treatment guidelines for adult WT, radical nephrectomy followed by adjuvant chemotherapy, with or without radiotherapy, is recommended [9].

## Conclusion

WT in adults is exceptionally rare and is often considered more aggressive than in children. However, recent evidence suggests that poorer outcomes may result from delayed diagnosis and lack of codified treatment protocols. This stage II case challenges the assumption of a poor prognosis in adults, emphasizing the importance of early detection, accurate staging, and timely surgical intervention. Such management may yield outcomes similar to pediatric cases, highlighting the need for increased clinical awareness and standardized treatment.

## Data Availability

No new data were generated or analyzed in support of this research.
